# Supplementary material for: Optimized methods for the targeted surveillance of extended-spectrum beta-lactamase-producing Escherichia coli in human stool
Source: Microbiol Spectr. 2024 Nov 22;13(1):e01058-24. doi: 10.1128/spectrum.01058-24 (PMC11705872; doi:10.1128/spectrum.01058-24)
Supplement: Supplemental figures — Fig. S1 to S3. [file spectrum.01058-24-s0001.docx]

| 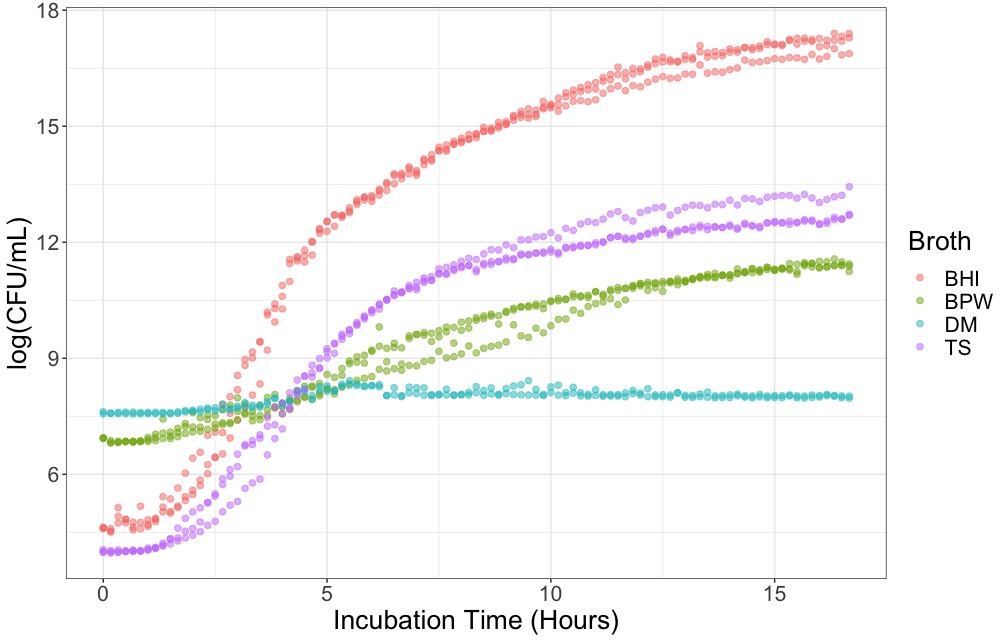 |
| --- |
| **Supplementary Figure 1** Growth of reference (NCTC 13441) ESBL-producing E. coli in different pre-enrichment broths (Brain-Heart Infusion (BHI), Buffered Peptone Water (BPW), Davis Minimal (DM) and Tryptic Soy (TS)) measured over 24 hours |

| 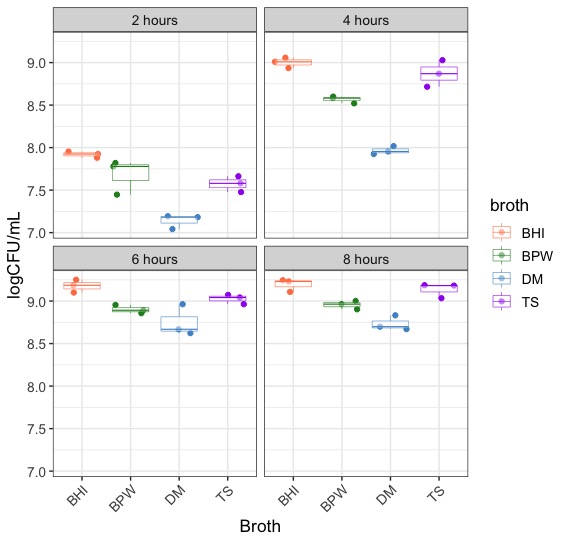 |
| --- |
| **Supplementary Figure 2** Quantitation of reference (NCTC13441) ESBL-producing E. coli after different incubation times (2, 4, 6 and 8 hours), in four different pre-enrichment broths (Brain-Heart Infusion (BHI), Buffered Peptone Water (BPW), Davis Minimal (DM) and Tryptic Soy (TS)) |

***
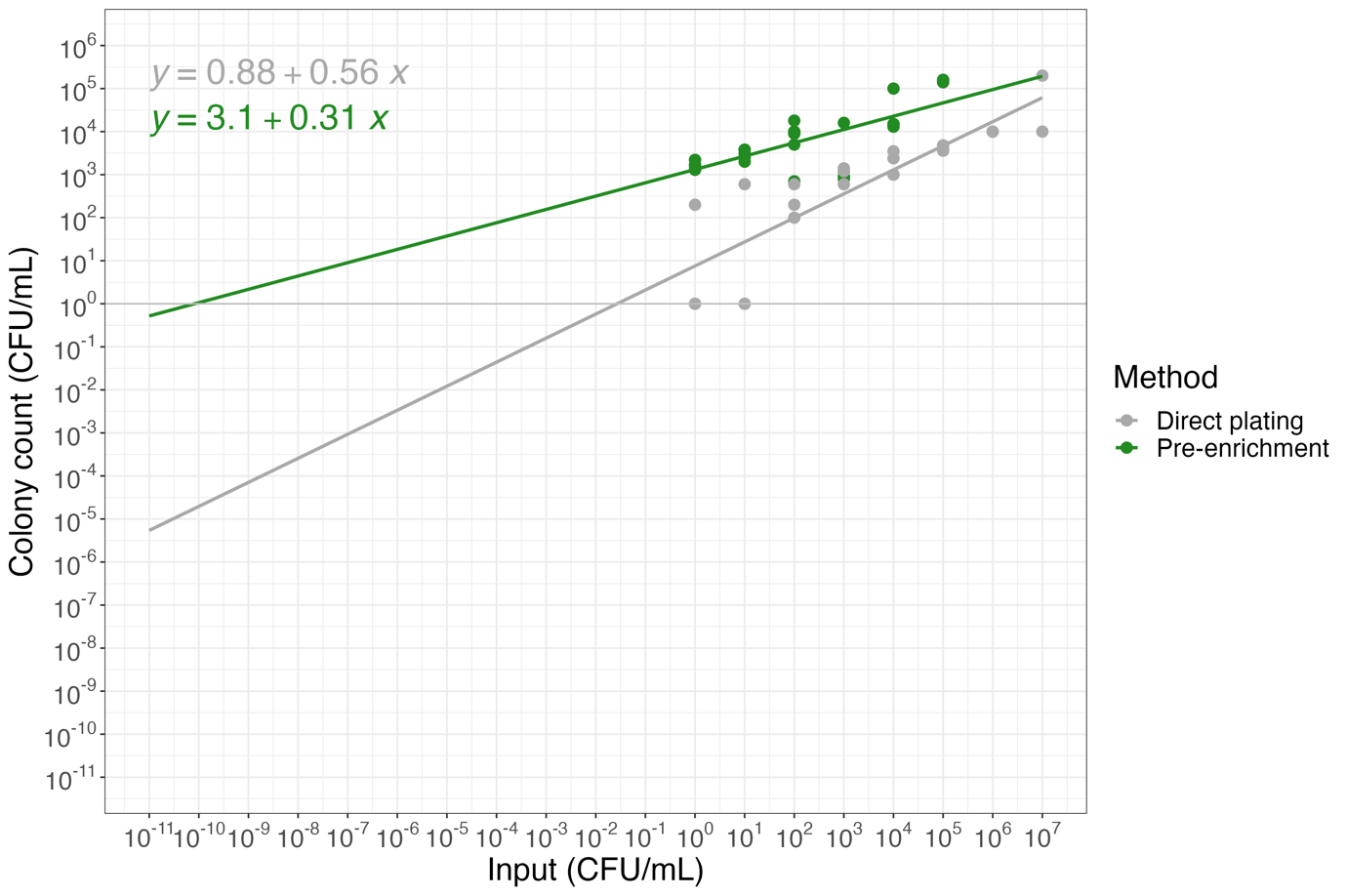
***

***Supplementary Figure 3*** *Colony counts for different input concentrations of a clinical ESBL-EC strain (CAB17W) spiked into stool and directly plated on cefotaxime supplemented MacConkey or pre-enriched before plating*
